# Single Molecule Analysis of c-*myb* Alternative Splicing Reveals Novel Classifiers for Precursor B-ALL

**DOI:** 10.1371/journal.pone.0022880

**Published:** 2011-08-11

**Authors:** Ye E. Zhou, John P. O'Rourke, Jeremy S. Edwards, Scott A. Ness

**Affiliations:** Department of Molecular Genetics and Microbiology, University of New Mexico Health Sciences Center, Albuquerque, New Mexico, United States of America; University of Florida, United States of America

## Abstract

The c-Myb transcription factor, a key regulator of proliferation and differentiation in hematopoietic and other cell types, has an N-terminal DNA binding domain and a large C-terminal domain responsible for transcriptional activation, negative regulation and determining target gene specificity. Overexpression and rearrangement of the c-*myb* gene (MYB) has been reported in some patients with leukemias and other types of cancers, implicating activated alleles of c-*myb* in the development of human tumors. Alternative RNA splicing can produce variants of c-*myb* with qualitatively distinct transcriptional activities that may be involved in transformation and leukemogenesis. Here, by performing a detailed, single molecule assay we found that c-*myb* alternative RNA splicing was elevated and much more complex in leukemia samples than in cell lines or CD34+ hematopoietic progenitor cells from normal donors. The results revealed that leukemia samples express more than 60 different c-*myb* splice variants, most of which have multiple alternative splicing events and were not detectable by conventional microarray or PCR approaches. For example, the single molecule assay detected 21 and 22 splice variants containing the 9B and 9S exons, respectively, most of which encoded unexpected variant forms of c-Myb protein. Furthermore, the detailed analysis identified some splice variants whose expression correlated with poor survival in a small cohort of precursor B-ALL samples. Our findings indicate that single molecule assays can reveal complexities in c-*myb* alternative splicing that have potential as novel biomarkers and could help explain the role of c-Myb variants in the development of human leukemia.

## Introduction

The c-*myb* gene (MYB) encodes a transcription factor that is absolutely required for normal hematopoiesis [1] including the differentiation of stem cells into committed progenitors [2], the normal development of myeloid and erythroid lineages [1], [3] and for B-cell and T-cell differentiation [4], [5]. (Note: we use the designations c-*myb* and c-Myb for the gene and protein, respectively, and to distinguish the normal cellular versions from the oncogenic variants v-*myb* and v-Myb) The c-Myb protein has a highly conserved DNA binding domain near its N-terminus and a large C-terminal domain required for transcriptional activation as well as negative regulation [6]–[8]. In addition, mutations or modifications of c-Myb can change its activity, converting the normal regulator into a potent oncoprotein that transforms immature myeloid cells in tissue culture and induces leukemias in animals [9]. Activated alleles of the c-*myb* gene have been identified in numerous human malignancies, including several types of leukemias as well as colon, breast and head and neck tumors [10]–[12], suggesting that c-*myb* is an important human oncogene [13]. However, the mechanisms leading to activation of the c-*myb* gene, and its conversion from a normal regulator to an oncogene, remain largely unexplained [13].

The activities of c-Myb are conflicting, affecting both differentiation and proliferation, and relatively minor changes can completely change its activity and specificity. For example, individual point mutations change the ability of c-*myb* to regulate specific target genes [14], [15]. Furthermore, microarray studies have shown that c-Myb regulates totally different sets of target genes than v-Myb, a truncated, mutated and oncogenic allele of c-Myb encoded by Avian Myeloblastosis Virus [16]. Most of the differences in the activity of v-Myb are due to changes in the large C-terminal domain, which appears to control target gene specificity by affecting protein-protein interactions [8], [17]. The implication is that the activity of c-Myb is highly variable and subject to change through mechanisms that alter its C-terminal domain, such as post-translational modifications, mutations or deletions. Such modifications can target c-Myb to different promoters and convert it from a normal regulator into a potent transforming protein.

One of the mechanisms affecting the C-terminal domains of c-Myb is alternative RNA splicing [13]. The human c-*myb* gene is located on chromosome 6q22-q23 and spans 15 exons plus 6 alternative exons. Interestingly, all of the alternative exons are in the region of the gene encoding the C-terminal domains that affect target gene specificity. Alternatively spliced c-*myb* transcripts have been detected in both humans and animals and in numerous tissues [18]–[22] and some c-*myb* splice variants with altered C-terminal domains have increased transforming activities [22]–[24]. Our own studies showed that alternative splicing of c-*myb* is tightly regulated during the differentiation of primary human hematopoietic progenitor cells and that levels of some alternatively spliced c-*myb* transcripts are elevated in leukemia samples compared to normal bone marrow cells [18]. Alternative splicing could be a whole new mechanism of unmasking the oncogenicity of c-*myb* and could play an important role in leukemogenesis.

The conventional way to monitor alternative splicing variants is by using real-time PCR or microarray assays that measure the expression of individual exons or individual exon junctions. However, these methods are not able to detect multiple changes that might occur in the same alternatively spliced mRNA, such as the addition of two alternative exons. The presence of upstream alternative exons could change the nature of the protein that is produced, with different biological consequences. To circumvent this problem, we enlisted a single molecule assay based on polonies or PCR colonies [25] to analyze c-*myb* RNA splicing patterns in normal cells, cell lines and a small cohort of pediatric precursor B-ALL patient samples. Our results show that alternative splicing of c-*myb* RNAs is highly complex and that leukemias produce elevated levels of c-Myb splice variants, implicating c-*myb* alternative splicing in leukemogenesis and transformation.

## Results

### Alternative splicing of c-myb transcripts is complex and combinatorial

**Figure 1:**
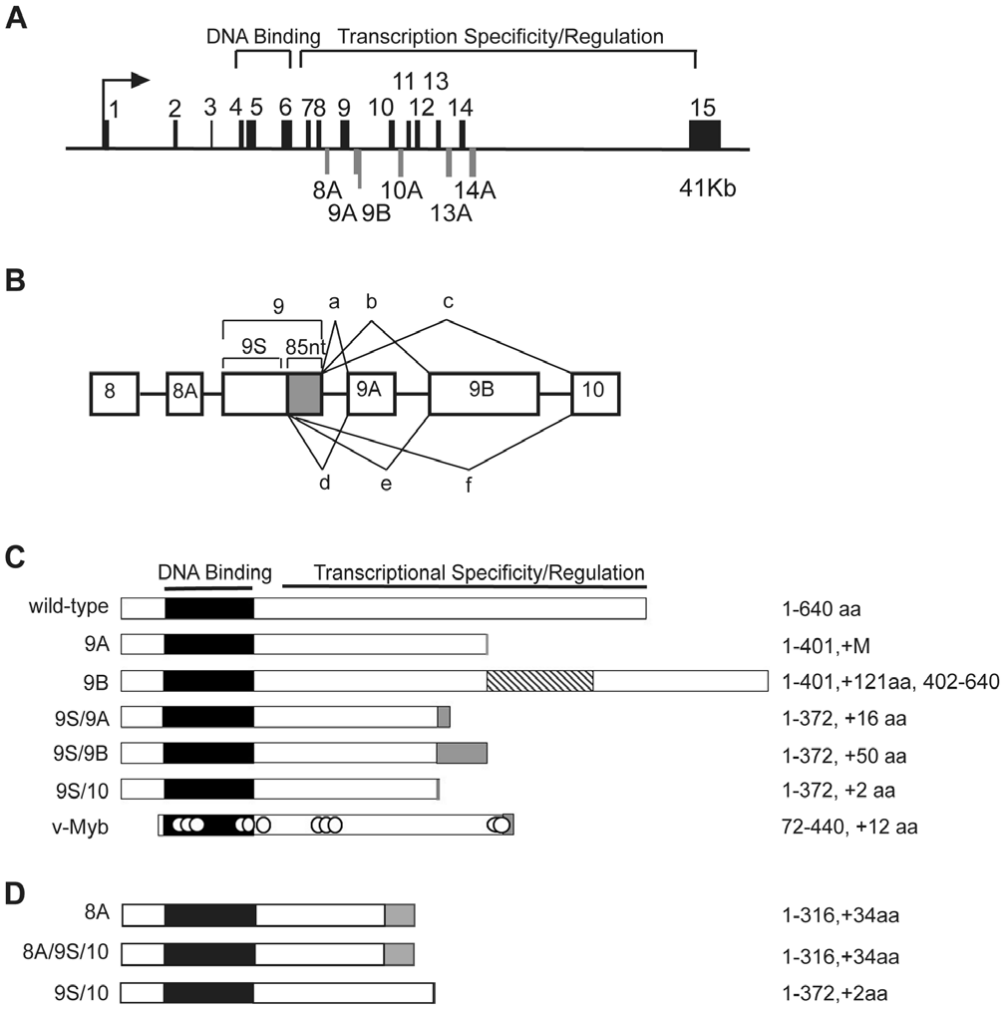
Alternative splicing in the c-*myb* gene. (A) Structure of the human c-myb gene. The diagram shows the exon structure of the human c-myb gene, which spans more than 41,000 nucleotides. The arrow marks the major transcription initiation site and exons 1–15, which are included in the wild type version of c-Myb, are indicated above the line. The highly conserved DNA binding domain of c-Myb is encoded by exons 4–6, and the C-terminal domains that control transcriptional activity and specificity are encoded by exons 7–15, as indicated. The latter region also includes the 6 alternative exons, shown below the line. (B) Alternative splicing in exons 8–10. A cryptic splice donor site in exon 9 generates a short (9S) version lacking 85 nt (shaded). Both the long and short forms of exon 9 can be spliced to alternative exons 9A or 9B or to exon 10. (C) Diagrams of wild type c-Myb and the 9A, 9B, 9S/9A, 9S/9B and 9S/10 splice variants. The structure of the v-Myb protein encoded by Avian Myeloblastosis Virus is shown for comparison. The highly conserved Myb DNA binding domain is shaded black. The variant proteins lack the normal C-terminus of c-Myb and have unique regions, indicated by shaded boxes. The numbers on the right summarize the structures of the proteins with the included amino acids (aa) and the changes relative to wild type c-Myb. (D) Structures of the proteins encoded by splice variants 8A, 8A/9S/10 and 9S/10. These variants are described in the text. Labels and numbering are as in panel (C).

The transforming activity of c-Myb has often been linked to deletions or mutations in its C-terminal domains [13], which can lead to the regulation of different sets of target genes [8], [16], [17]. Our previous studies showed that alternative splicing of c-*myb* transcripts is highly regulated in hematopoietic cell differentiation and in leukemias, and could lead to the production of variant c-Myb proteins with different C-terminal domains [18]. We set out to determine whether the shift in the patterns of c-*myb* alternative splicing could be a novel mechanism for activating c-Myb oncogenicity in leukemias. However, we were faced with a complication due to the complexity and combinatorial nature of c-*myb* alternative RNA splicing. As shown in Figure 1A, the human c-*myb* gene spans 41 kb on chromosome 6q and contains 15 exons that compose the wild type transcript, plus 6 alternative exons (8A, 9A, 9B, 10A, 13A and 14A). The complexity is increased further by exons that have multiple splice donor sites. For example, as shown in Figure 1B, exon 9 can also be spliced as an 85 nucleotide shorter “9S” version. The normal exon 9 and the short 9S form can be joined to exons 9A, 9B or 10 (labeled a, b, c and d, e, f, respectively), so alternative splicing of just this one exon produces 6 different splice variants. All six variants encode different versions of the c-Myb protein, with identical N-terminal DNA binding domains (shaded black in Figure 1C), but different C-terminal domains that affect transcriptional activity and that control which target genes get regulated [8], [16], [18], [26]. Analysis of chicken and mouse bone marrow has shown that the use of the 9S splice site is conserved in other vertebrate species, suggesting that it is an integral and important part of the c-*myb* gene (Figure S1). For simplicity, we designate the 9S exon spliced to exon 10 as the 9S/10 splice variant, and so on. Additional complexity is introduced by other alternative exons.

Our previous studies [18] also identified a large number of transcripts containing more than one alternative splice event, e.g. the inclusion of exon 8A in a splice variant that also has exon 9S spliced to exon 10 (Figure 1B). These combinatorial transcripts are problematic, because the usual methods of measuring alternative splicing do not assess them correctly. For example, a real-time PCR (QPCR) assay or exon junction microarray measuring the 9S/10 splice junction would not distinguish whether exon 8A was also present in the same transcript, but exon 8A contains a stop codon so all transcripts containing 8A produce the same truncated protein (Figure 1D) regardless of the splicing events affecting exon 9. Almost all c-*myb* alternative splicing occurs in the exon 6–11 region, which is too long to be analyzed using currently available next-generation DNA sequencing technologies. Therefore, in order to determine what c-Myb protein products were being produced in different samples, we had to find a quantitative methodology that would determine the levels of expression as well as the structures of the c-*myb* transcripts: an assay that would analyze the structures of single and intact c-*myb* mRNA molecules.

### Detection of c-myb alternative splicing variants using a polony-based assay

**Figure 2:**
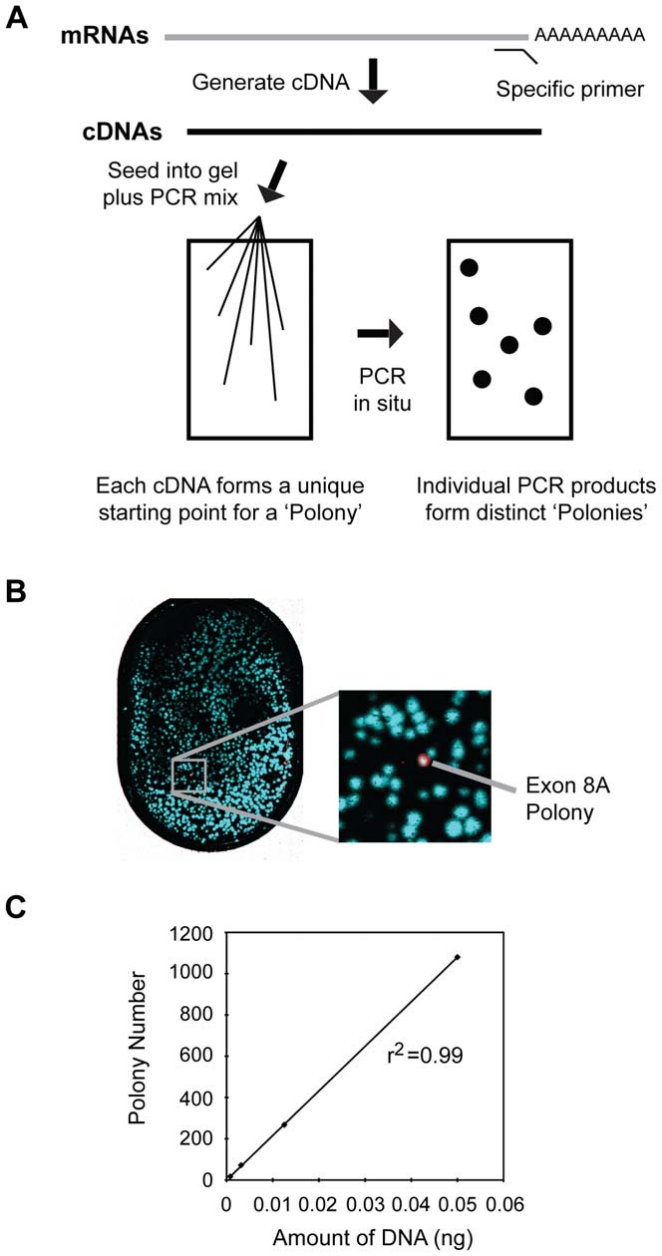
Sensitivity and linearity of the polony assay. (A) Outline of polony assay. Gene-specific primers are used to convert mRNA to cDNA, which is then seeded into a thin acrylamide gel containing primers, enzymes and other components necessary for in situ PCR, which results in the formation of “PCR colonies”, or polonies. The polonies are fixed in the gel, but can be interrogated by sequential hybridization with specific fluorescently labeled oligonucleotides. (B) Sensitivity of polony assay. Plasmids containing cDNAs for wild type c-myb or the 8A splice variant were mixed in a ratio of 5,000∶1. After in situ PCR, the slide was hybridized sequentially with fluorescently labeled probes specific for exon 8, exon 8A or exon 11. The full polony slide is shown at left and the enlargement at right shows a single detected exon 8A-containing splice variant (white). In the false color image the polonies containing only exons 8 and 11 appear Cyan in color, the polony containing all three exons appears white. (C) Linearity of polony assay. Different amounts of wild-type c-myb plasmid were used as template in the polony assay and the number of polonies detected with the exon 11 probe was plotted against amount of plasmid template used in the assay. The assay was very linear, as shown by the correlation coefficient r2, which was 0.99.

To produce a quantitative and complete picture of c-*myb* alternative splicing, we adopted a single molecule analysis procedure utilizing polonies, or PCR colonies [25]. In the polony procedure (Figure 2A), mRNA samples are converted to cDNA by reverse transcription, the mixture of individual cDNA template molecules are then seeded into and immobilized in a thin polyacrylamide gel containing primers and other reagents necessary for PCR amplification. In situ PCR is conducted directly in the gel, which prevents the PCR products from diffusing away, so that each template produces a “PCR colony”, or polony, each of which arose from the same template cDNA molecule. The structures of the polonies are then interrogated through sequential rounds of hybridization with fluorescently labeled, exon- or exon-junction-specific probes.

The Polony assay provides a distinct advantage over QPCR assays, which only measure one exon or one splice junction at a time and cannot determine which changes occur in the same RNA molecules. The polony approach can determine the structural map of the entire cDNA molecule that served as the polony template. This is critical for the analysis of c-*myb* alternative splicing, since different splice variants produce different proteins with potentially unique activities. The polony assay can distinguish these complex splice variants to more accurately quantify which versions of c-Myb protein would be produced. The QPCR or microarray approaches cannot distinguish a transcript that has only exon 10A from one that has both exons 8A and 10A, so it does not accurately predict which proteins will be produced.

To test the sensitivity of this assay on detecting a rare splice variant, we generated polonies from a region of the c-*myb* transcript spanning exons 6 to 11, where the majority of alternative splicing occurs. We used a mixture of wild type c-*myb* cDNA plus an alternatively spliced cDNA containing exon 8A added at different molar ratios (e.g. 5000∶1). After in-gel amplification to produce the polonies, multiple rounds of hybrifodization were performed sequentially with fluorescently labeled probes specific for different exons or splice junctions (Table S2). The microscope slide was scanned after each round of hybridization to record which polonies were positive for each probe. Since each polony was generated from a single template cDNA molecule and every polony had a fixed address on the polony slide, the sequential hybridization steps produced a series of images that were converted into an exon map for each polony or cDNA template. As shown in Figure 2B, the polony assay was highly sensitive and able to detect one transcript containing alternative exon 8A in a background of more than 5,000 wild type c-*myb* transcripts. Thus, the assay is well suited for detecting rare splice variants in a background of wild type transcripts. The linearity of the polony assay was examined by seeding different amounts of wild type c-*myb* plasmid templates. As shown in Figure 2C, the polony assay was linear over a wide range of concentrations (r^2^ = 0.99). Thus, the polony approach provides a sensitive and linear methodology for analyzing the structures and relative expression levels of alternatively spliced c-*myb* transcripts, and it yields more structural information than conventional QPCR approaches.

To test the polony technique using more relevant samples, we examined the expression of c-*myb* transcripts in 2 human hematopoietic cell lines (K562 cells, Jurkat cells) and 2 types of primary cells (peripheral blood leukocytes or PBLs and CD34+ progenitor cells) that were previously analyzed extensively for c-*myb* splice variants by QPCR and shotgun cloning and sequencing [18]. The four cell types expressed wild type (WT) plus 10 additional splice variants of c-*myb*, some of which have not been previously described (Table 1). The patterns of c-*myb* splice variants identified by the polony assay were complex, demonstrating the effectiveness of this assay to detect multiple splice events in a single transcript. For example, the 9B/10A splice variant, an alternatively spliced c-*myb* transcript containing both alternative exons 9B and 10A, was detected in both K562 (0.53%) and Jurkat (1.07%) leukemic cell lines, but not in two primary human hematopoietic cell samples. The 9S/10/10A splice variant, which includes the short (9S) version of exon 9 spliced to exon 10, plus an alternative exon 10A, was detectable in Jurkat (0.36%) and CD34+ cells (0.09%) but not in the other cell types. Approximately 10%–15% of the c-*myb* transcripts in these samples were alternatively spliced variants and the polony assay detected levels of alternative exons 8A, 9A, 9B and 10A that were similar to the results obtained previously using either QPCR or direct shotgun sequencing techniques [18]. We conclude that the polony assay provides quantitative information about c-*myb* alternative splicing that rivals or exceeds the results obtained using QPCR, and that it offers a valuable and powerful alternative for quantifying transcripts with complex structures.

**Table 1:** Expression of c-myb splice variants in cell lines and primary cells.

Description	Accession No.	Exontyping	K562	Jurkat	PBL	CD34+
Del9	AY787448	6-7-8-10-11	ND	1.78%	ND	0.26%
Del8	AY787447	6-7-9-10-11	1.33%	ND	1.05%	0.45%
9S/10	AY787470	6-7-8-9S-10-11	2.13%	0.36%	1.83%	0.89%
Del10	NA	6-7-8-9-11	0.27%	ND	ND	0.09%
Del8/9A	NA	6-7-9-9A-10-11	0.27%	ND	ND	ND
9A	AY787464	6-7-8-9-9A-10-11	0.54%	0.36%	0.52%	0.18%
9B	AY787467	6-7-8-9-9B-10-11	6.91%	5.69%	2.09%	2.09%
9B/10A	NA	6-7-8-9-9B-10-10A-11	0.53%	1.07%	ND	ND
9S/10/10A	AY787471	6-7-8-9S-10-10A-11	ND	0.36%	ND	0.09%
10A	AY787450	6-7-8-9-10-10A-11	2.66%	4.63%	6.54%	2.84%
8A	AY787454	6-7-8-8A-9-10-11	1.06%	1.43%	3.40%	3.76%
WT	AY787475	6-7-8-9-10-11	84.57%	84.34%	84.55%	89.35%

Expression of c-*myb* alternative splicing isoforms measured by polony assay and expressed as percentage of the total detected c-*myb* transcripts. ND: not detected. NA: not available. Since these products were only detected using the polony assay, their complete nucleotide sequences have not been determined and accession numbers are not available.

### Comparison of the polony and QPCR assays

**Figure 3:**
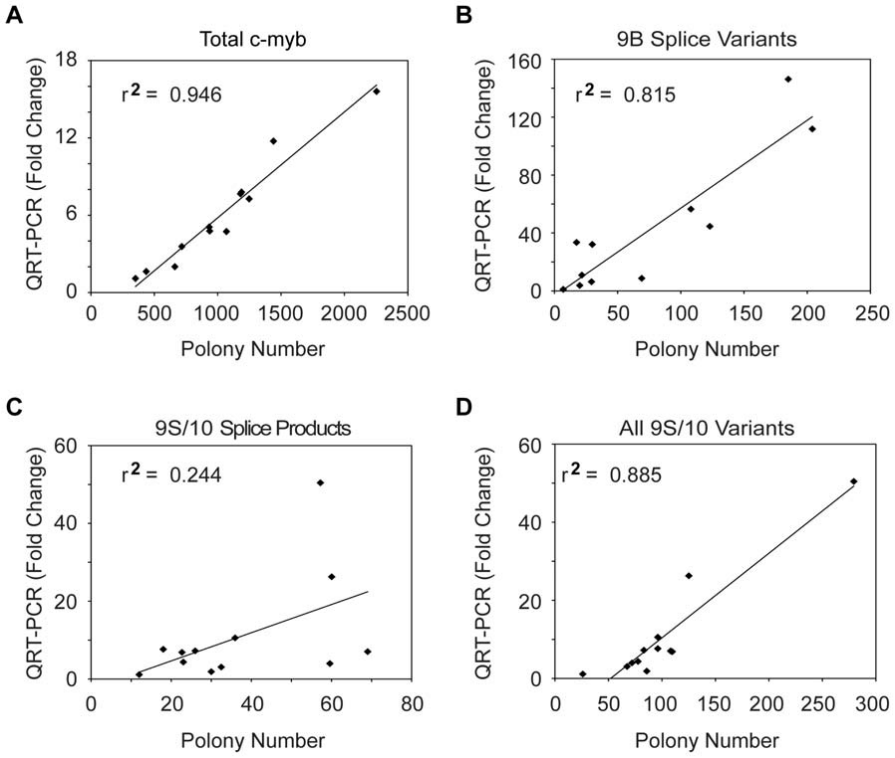
Comparison of exon-specific (QPCR) to single molecule (polony) assays. Scatter plots compare the levels of different c-myb splice variants detected by bulk QPCR or single molecule polony-based assays for (A) total c-myb transcripts or (B–C) the 9B or 9S/10 splice variants, respectively. The QPCR and polony assays agree nicely (r2 = 0.946) for total c-myb transcripts, but the results for the 9B variant agree only modestly (r2<0.82) and the results for the 9S/10 variant did not correlate well (r2<0.25). (D) Compiling the polony data by combining all the detected transcripts that contain the 9S/10 exon produces data that correlate much better (r2>0.88) with the QPCR data. Thus the differences in the two assays are due to the exclusion of variants in the polony results that are predicted to produce different protein products, which are detectable in the polony assay but not by QPCR.

The results described above demonstrated the usefulness of the polony assay for following patterns of alternative RNA splicing and suggested that it could provide different and more extensive information than QPCR. Like all leukemias [13], precursor B-ALL samples express c-*myb*, but changes in total levels of c-*myb* expression detectable in microarray or QPCR assays are not correlated with differences in prognosis or outcome [27]. We next applied both QPCR and the polony assay to monitor c-*myb* expression and alternative splicing patterns in a small cohort (n = 13) of pediatric precursor B-ALL patient samples, with the goal of determining whether changes in c-*myb* alternative splicing could be a useful biomarker. RNA samples from each patient were converted to cDNA, which was used for QPCR assays or used to seed the polony assays, which were interrogated by probes for various c-*myb* exons. Up to 1500 polonies per slide, or 3000 polonies in two replicate measurements, were queried for each patient sample. As shown in Figure 3A, the QPCR and polony assay results for the total c-*myb* transcripts in the patient samples correlated quite well (r^2^ = 0.946), suggesting that both assays were similarly efficient and were equally linear. This confirms the results described above showing that both the QPCR and polony approaches are very sensitive and provide quantitative results, at least for measuring the total levels of c-*myb* expression.

However, the differences between the two methods were more dramatic when they were used to measure the expression of individual c-*myb* splice variants. As shown in Figure 3B, the QPCR and polony assays agreed only modestly (r^2^ = 0.815) when they were used to assay the levels of the 9B splice variant. The situation was much worse for the 9S/10 splice variant (Figure 3C), where the results from the assays were poorly correlated (r^2^ = 0.244). Thus, although the two methods gave highly similar results for the total expression of c-*myb*, they gave quite different results for some splice variants, such as the 9S/10 variant.

The differences in the QPCR and polony assay results for individual splice variants can be explained when the details of the polony results are scrutinized (Table S3). While QPCR measures only the relative abundance of each exon, such as 9B, or each splice junction, such as 9S/10, the polony assay measures the structure of each mRNA molecule and the data are converted into predicted protein structures. Consequently, in the polony assay data, some variants are excluded from the count or counted as a different variant if they would give rise to a different protein product. As shown in Table S3, the polony assay detected a total of 21 different splice variants that contained exon 9B, only one of which is predicted to produce the 9B splice variant protein, which has a novel 121 amino acid in-frame insertion at position 402 (abbreviated: 1–401, +121aa, 402–640). Similarly, the polony assay detected 22 different splice variants that included the short 9S exon, only two of which produce the 9S/10 protein (1–372, +2aa). Thus, the QPCR assay overestimates the number of splice variants that will produce the 9B and 9S/10 protein products because it fails to take into account the other changes in the transcripts. Indeed, if the polony assay data are compiled in order to simulate the QPCR assay results by pooling all the enumerated splice variants that contain the 9S/10 splice junction, the results of the two assays again correlate quite well (r^2^>0.88, Figure 3D). Thus, the polony assay detects all the different splice variants containing specific exons and provides a more detailed glimpse of the spectrum of proteins that are predicted to be produced as a consequence of alternative RNA splicing.

### Alternatively spliced variants of c-*myb* in pediatric B-ALL

**Figure 4:**
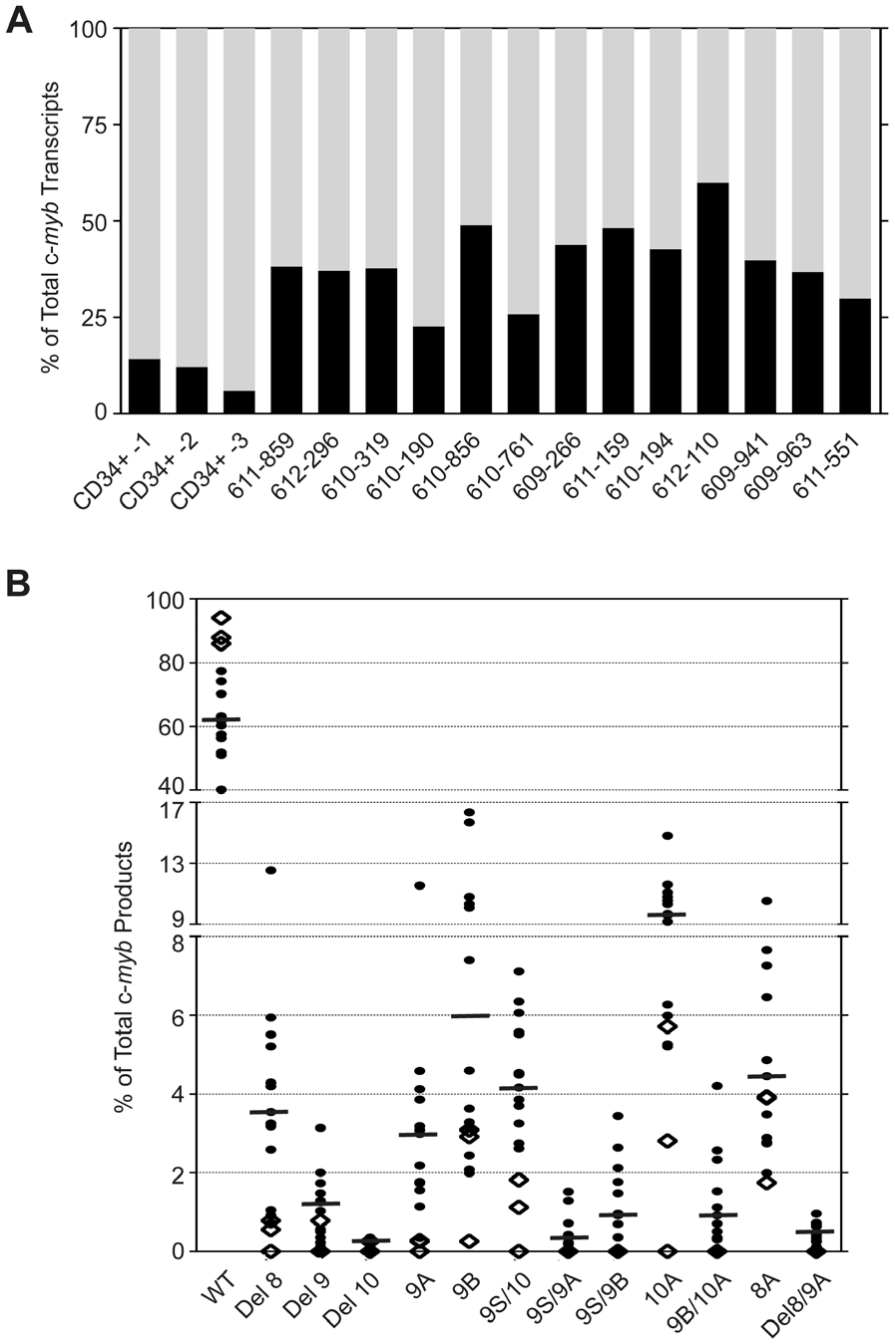
Alternative splicing of c-*myb* transcripts in normal and leukemic cells. (A) Rates of c-myb alternative splicing in normal cells and leukemias. Polony assays were used to measure the levels of normal and alternatively spliced c-myb transcripts in normal CD34+ progenitors from healthy donors (n = 3) or in pediatric precursor B-ALL patient samples (n = 13). The black and gray sections of each bar indicate the fractions of alternatively spliced or wild type transcripts, respectively. (B) Patterns of c-myb alternative splicing in pediatric precursor B-ALL samples. Dots indicate levels of wt or splice variant expression, as a fraction of total c-myb transcripts detected, as determined by polony assay for pediatric precursor B-ALL samples (n = 13, filled circles) or CD34+ cells from normal donors (n = 3, empty diamonds). Each symbol represents the average of duplicate measurements and the horizontal bars represent the median values for the leukemia samples.

Using the polony results (Table S3), we compared the total levels of wild type and alternatively spliced transcripts in the leukemia samples, which revealed that c-*myb* alternative splicing was dramatically increased in the leukemias compared to normal CD34+ cells. In 3 normal donor samples (progenitor CD34+ cells), the combined expression level of c-*myb* splice variants ranged from 5%–15% of the total c-*myb* transcripts (Figure 4A). However, the splice variants were much more abundant in the 13 pediatric leukemia patient samples, accounting for 25% to 60% of the total c-*myb* transcripts, suggesting that alternative splicing of c-*myb* transcripts occurs much more frequently in leukemias than in normal hematopoietic cells. Indeed, alternative splicing in all of the leukemia samples was more than twice the median level observed in the normal CD34+ cell samples.

The polony assay detected 61 different splice variants of c-*myb* in the leukemia samples (Table S3). They can be categorized as: (a) exon deletions, in which one or more exons are skipped (for example, Del8; Del9; Del10; Del8/Del9; Del8/9/Del10); (b) short exons produced through the use of cryptic splice sites (for example, the 9S exon lacking 85 nucleotides); (c) inclusion of one or more alternative exons (for example, 8A; 9A; 9B; 10A); or (d) combinations of these splicing events (for example, 9S/9A; Del8/9S/9B; Del8/9/9B/10A; 9B/10A; 8A/10A). Some of these variants are predicted to encode the same variant c-Myb proteins, since the introduction of a premature termination codon is encoded by the upstream alternative exon(s) (8A, 9A, and 10A). Truncation of the C-terminus of c-Myb activates its leukemogenic activities [6], [28], so these variant proteins could be expected to have increased transforming activities. The C-terminus of c-Myb protein also contains the major site of ubiquitination, and truncated variants are more stable [29], so the shorter proteins produced by the splice variants could also be expected to be more stable than full length, wild type c-Myb protein.

The expression levels of the 13 most abundant splice variants in the normal CD34+ and leukemia samples, each accounting for more than 1% of the total c-*myb* transcripts, are shown in Figure 4B. The results revealed highly variable levels of each of these splice variants amongst the patient samples. For example, the levels of the 9S/10 transcript varied several fold in these 13 patients, from about 1% to more than 7% of the total. The level of the 9B variant ranged from 2% to 17% of the total, and the 10A transcript varied from 5% to 15% of the total. On average, the splice variants were far more abundant in patient samples than in normal CD34+ cells. In addition, the splicing repertoires of c-*myb* were distinct in patient samples compared to healthy CD34+ cells and each patient displayed a unique pattern of splice variants: some patients expressed more than 25 c-*myb* isoforms, while others only expressed 12 splice variants (Table S3). Taken together, these data confirm that overall alternative splicing of the c-*myb* gene was significantly elevated in leukemia samples compared to normal CD34+ cells, suggesting that alternative splicing of c-*myb* could provide a novel biomarker linked to prognosis or patient outcome.

### Alternative splicing of c-*myb* as a potential prognostic marker

**Figure 5:**
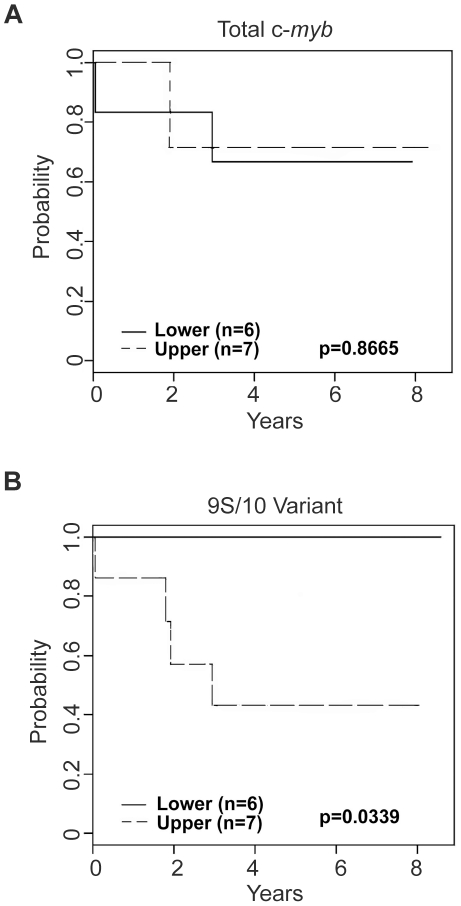
Survival plots for patients grouped by c-*myb* variant expression levels. Precursor B-ALL patients (n = 13) were grouped according to their expression above (dotted line) or below (solid line) the median expression level for (A) total c-myb transcripts or (B) the 9S/10 variant. The plots show overall survival curves for the high expression or low expression groups of leukemia samples, with p-values as indicated.

The higher rates of alternative splicing in leukemias suggested that expression of particular c-*myb* splice variants could be a potential prognostic signature. Analysis of the alternative splicing data (Table S3) showed that only wild type c-Myb and 5 splice variant proteins: Del8, 8A, 9S/10, 9A and 10A were expressed in all the leukemia samples. By analyzing the levels of expression in leukemias and normal samples (Figure 4B) we identified three splice variants: Del8, 9A and 9S/10 that were consistently expressed more highly in the leukemias than in the normal CD34+ progenitor cells. Next, we used the polony exon profiling data to group the 13 patients based on whether they expressed above or below the median level of each of the most commonly expressed splice variants. The survival data for these patients were then analyzed to see if the groupings, based on high or low splice variant expression in the leukemia samples, correlated with better or worse outcome. As shown in Figure 5A, the patients displaying the highest and lowest levels of total c-*myb* transcripts displayed similar survival curves, indicating that the absolute level of c-*myb* expression, as might be measured in microarray assays or by conventional PCR approaches, did not correlate with outcome in this context. Similar results were obtained for the majority of the splice variants analyzed (not shown). However, the groups of patients with high and low levels of expression of the 9S/10 splice variant, which is generated through the use of an alternative splice donor site in exon 9 and which encodes a truncated version of c-Myb protein (Figure 1C), were quite striking. As shown in Figure 5B, the patients with below median expression of variant 9S/10 all survived at least 8 years (solid line), while the patients with above median expression of 9S/10 had significantly lower survival (p<0.05). This result was not observed when the QPCR levels of 9S/10 were used in the analysis, showing that the single molecule polony-based assay detects more complexity in the splicing patterns, and can reveal some patterns that correlate with outcome. Although these results are from a small cohort of patients, they suggest that increased levels of the 9S/10 splice variant could be a negative prognostic factor for pediatric precursor B-ALL and that analysis of c-*myb* alternative splicing could prove to be a useful prognostic marker for leukemias such as precursor B-ALL.

## Discussion

The c-*myb* gene is organized in way that the vast majority of alternative RNA splicing occurs in exons encoding the C-terminal domains of c-Myb protein, generating a family of transcription factors with the same N-terminal DNA binding domains but differences affecting transcriptional activity, negative regulation and target gene selection [8], [16]–[18]. When we adapted a polony assay to the analysis of c-*myb* alternative splicing, we detected much more complex and combinatorial alternative splicing events than could be monitored using microarray or QPCR methods. We found that c-*myb* alternative splicing is greatly increased in precursor B-ALL samples, that the pattern of c-*myb* alternative splicing is more complex in leukemia patients than in normal CD34+ cells, and that the pattern is distinct in each patient sample, suggesting that activation of alternative RNA splicing is a mechanism that could contribute to leukemogenesis. By analyzing a small cohort of patient samples, we identified a splice variant, 9S/10, whose expression correlated with poor survival and which accounted for up to 7% of the total c-*myb* transcripts in leukemia samples. Although confirmation will require detailed analyses of larger patient cohorts, the results are intriguing and suggest that patterns of c-*myb* alternative splicing have the potential to provide an independent means of classifying patients into different treatment groups, similar to gene expression profiling [30]. Interestingly, the latter approach compares total levels of mRNAs, but our analyses showed that total c-*myb* levels did not correlate with patient survival, while levels of some individual splice variants did (Figure 5). This may explain why microarray assays did not identify c-*myb* as a component of the classifier distinguishing high-risk patient groups, even though c-Myb is a key regulator of proliferation and differentiation in hematopoietic and other cells [9], [13].

A key difference in our study compared to previously reported analyses of alternative splicing is our use of a single molecule polony assay to analyze the various c-*myb* transcripts. Unlike microarrays or QPCR, which only measure the relative levels of expression of specific exons or splice junctions, the polony assay provides structural information over the entire length of the template cDNA. Thus, the polony assay can identify transcripts that contain both an upstream variation, such as inclusion of exon 8A, and also a downstream variation, such as inclusion of exon 9B. The other methods can detect each of these events but cannot determine whether both splice variations are present in the same, or in different mRNA molecules. In this example, since exon 8A introduces a premature stop codon, mRNAs that include exon 8A either alone or in combination with exon 9B produce the same truncated protein. Thus, the polony assay is better able to predict which types of variant c-Myb proteins will be produced in a given sample. At present, even next-generation sequencing methods are limited to reading less than 1 kb of DNA sequence per molecule, so they are insufficient to determine the structure of the entire region of the c-*myb* transcript that becomes altered by alternative RNA splicing. Until next-generation methods are developed that can determine the structures of entire cDNAs, single molecule approaches such as the polony assay will remain the best alternative for following complex changes in alternative splicing. Unfortunately, the polony assay is rather cumbersome and is not suited to screening large numbers of patient samples, so improved assays will be required to extend these results to larger cohorts.

There are several reasons why analyzing c-*myb* RNA splicing patterns could be a useful classifier for leukemia samples. First, the analysis is extremely sensitive, requiring only a few nanograms of RNA for each assay. This means that the analysis of RNA can be performed without requiring the use of an additional bone marrow sample. In contrast, analyzing the expressed proteins directly, for example using Western blot assays, would require large numbers of cells, meaning additional bone marrow samples would have to be collected from the leukemia patients, which is especially problematic for pediatric patients. In addition, most of the proteins that are expected to be produced by the splice variants we studied lack any unique epitopes that could be detected in immuno-blots or by immunohistochemistry methods. For example, the 9S/10 splice variant is truncated but has only two novel amino acids added at its C-terminus. Although we did not analyze the expression of the protein products in the leukemia patient samples, there is reason to believe that most or all of the alternatively spliced c-*myb* transcripts are translated into proteins. In a previous study using cell lines and normal cells, we showed that all of the major c-*myb* splice variants were present in the cytoplasmic fraction of RNA, were polyadenylated and were bound to polysomes, suggesting that they are translated [18]. Finally, even if the alternatively spliced transcripts are not transported to the cytoplasm or are not translated into proteins, they can still serve as useful biomarkers if their levels correlate with prognosis or survival. Even if the expected c-Myb variant proteins are not produced, the alternatively spliced transcripts could serve as indicators of oncogene-activated signaling pathways that affect the RNA splicing machinery or even of aberrantly activated splicing factors, some of which have oncogene activity [31]. Thus, although we have not shown that the variant c-Myb proteins are expressed in the leukemias, the sensitivity of the RNA-based assay makes it more useful than analyzing proteins and its usefulness as a biomarker is not diminished even if the proteins are not expressed.

An important question concerns why the alternative splicing patterns differ in leukemias compared to normal cells, and why they vary amongst different patient samples. There is little information about the regulation of alternative splicing in leukemias, although the levels of specific splicing regulatory factors are altered in tumors, can affect the alternative splicing of some genes, including c-*myb*, and can act as oncogenes in some situations [31]. Several types of regulatory pathways, including transcriptional and post-transcriptional regulation and signaling cascades leading to post-translational modifications can affect the activities of factors controlling alternative RNA splicing [32]. Many of these pathways can be modified in leukemias, which could lead to changes in splicing patterns. In addition, histone modifications can affect alternative RNA splicing [33], implicating important leukemia-associated oncogenes that affect histone modifications in the control of alternative splicing [34], [35]. Thus, the regulation of alternative splicing is a relatively unstudied area that could be affected by many of the known oncogene pathways and that could play an important role in tumor development or progression.

Perhaps the most profound result from our study is that leukemias express such a large fraction, in some samples up to 60%, of c-*myb* transcripts that are alternatively spliced products (Figure 4A). Thus, a large fraction, in some cases even a majority of the c-Myb proteins in these samples are likely to be variant forms, rather than the expected wild type c-Myb. Interestingly, the two leukemia cell lines we analyzed, K562 and Jurkat cells, were much more similar to normal cells than to the leukemia samples in this respect, since they only expressed 10–15% of alternatively spliced c-*myb* transcripts (Table 1). Neither of these cell lines was derived from a precursor B-ALL, so the high level of alternatively spliced products we observed in the leukemias could be specific to precursor B-ALL. Alternatively, the cell lines may be a poor model system for studying the expression of c-*myb* variants. Analysis of additional leukemia types will be necessary to distinguish these possibilities.

Overall, our study explored the possibility of quantitatively monitoring c-*myb* alternative splicing in different samples by polony assay. The increased c-*myb* alternative splicing events in leukemia patient samples and the survival analyses suggested a tight link between the regulation of alternative splicing, the production of variant c-Myb proteins and their possible roles in leukemogenesis. Further investigation of more patients using the polony assay or related techniques will be needed to shed light on the clinical value of following c-*myb* alternative splicing in leukemia samples, which could potentially be used as a unique type of biomarker.

## Materials and Methods

### Ethics statement

Patient samples were obtained from the Children's Oncology Group Cell Bank (Proposal #2002-06) and were collected after written informed consent was obtained from the patients or their parents/guardians, who gave specific permission for use in future research projects. The use of the samples was also approved by the Human Research Review Committee (Project #03-222) at the University of New Mexico Health Sciences Center. No patient identifier information was provided to the authors of this manuscript.

### Cells, tissue culture and patient samples

Human erythroleukemia K562 cells (CCL-243, ATCC, Manassas, VA) and human Jurkat T-cells (TIB 152, ATCC, Manassas, VA) were maintained in RPMI1640 medium (GIBCO, Carlsbad, CA) with 10% Fetal Bovine Serum (PAA, Morningside QLD Australia). Buffy coat blood samples were purchased from United Blood Services (Albuquerque, NM) and peripheral blood leukocytes were purified by Ficoll (Amersham, Piscataway, NJ) density centrifugation and cultured in Iscove's Modified Dulbecco's Medium (IMDM from GIBCO) with 10 U each of interleukin-2 (IL-2; PeproTech, Rocky Hill, NJ) and phytohaemagglutinin per ml for 4 days prior to RNA isolation. Cytokine-mobilized CD34+ cells (purchased from the Fred Hutchison Cancer Research Center Large-Scale Cell Processing Core) were cultured in IMDM supplemented with BITS serum substitute, IL-3 (20 ng/ml), IL-6 (20 ng/ml), Stem Cell Factor (100 ng/ml), and FLT-3 ligand (100 ng/ml) (all from Stem Cell Technology, Vancouver, Canada).

### RNA expression and structure assays

Total RNA was isolated using RNeasy mini kits (Qiagen, Valencia, CA), cDNA was synthesized using a first-strand cDNA synthesis kit (Invitrogen, Carlsbad, CA), SYBR green-based real-time PCR used a Maxima qPCR kit (Fermentas, EU) and Taqman probe-based QRT-PCR used Taqman Universal PCR Master Mix (Applied Biosystems/Life Technologies, Foster City, CA), according to the supplied protocols. PCR reactions were performed in quadruplicate using primers described in Table S1. The results of relative gene expression assays were normalized to the level of GAPDH, and the data were analyzed using the comparative threshold cycle method [36].

### Polony amplification

After reverse transcription (as described above), the polony amplification was performed as previously described [25]. Briefly, the three-step procedure involves application of the gel matrix on a slide, infusion of PCR reagents and in situ PCR in a Slide Cycling ‘16/16’ Dual PCR Block (MJ Research, MA). Gel mix [10 mM Tris-HCL pH 8.3, 50 mM KCL, 0.01% gelatin, 1.5 mM MgCl_2_, 6% acrylamide, 0.35% N-N′ diallyltartardiamide (DATD), 0.035% Bis-acrylamide, 1 µM acrydite modified primer (Table S2), 0.1% Tween-20 and 0.2% BSA] was freshly prepared, ammonium persulfate (APS) and Tetramethylethylenediamine (TEMED) were added to a final concentration of 0.087% each and 15 µm thick gels were poured on glass microscope slides that had been partially coated with Teflon masks (Erie Scientific), which served as spacers between the slides and glass cover slips (22 m×30 mm, Fisher Scientific). The gels were allowed to polymerize in a dedicated pre-PCR hood for 15 min. The slides were washed 20 min in water, dried under the hood for about 25 min and 23 µl of polony amplification mix [10 mM Tris-HCl pH 8.3, 50 mM KCl, 0.01% gelatin, 0.2% Bovine Serum Albumin, 0.1% Tween-20, 1.33 µl of 10 µM reverse primer (Table S2), 200 µM dNTP mix, 0.335 U Jumpstart Taq and desired amount of template] was applied to the slides and covered with a cover slip. The slides were covered with mineral oil utilizing adhesive incubation chambers (Secure Seals SA 500, GRACE Bio-Labs), then cycled in a PTC-200 twin tower thermocycler as follows: denaturation (3 min at 94°C, amplification for 48–52 cycles (30 sec at 94°C, 45 sec at 62°C, 3 min at 72°C), extension (6 min at 72°C), chill to 4°C. After cycling, the slides were rinsed in hexane to remove the mineral oil.

### Denaturation and hybridization

After polony amplification, the cover slips were removed and the unattached strands of DNA were removed by incubating the slides in pre-warmed denaturing buffer (70% formamide, 1XSSC) at 70°C for 15 min. The slides were subsequently washed in water for 3 min, 2×4 min each in wash buffer 1E (10 mM Tris-HCL pH 7.5, 50 mM KCL, 2 mM EDTA, 0.01% Triton X-100) and 90 µl of annealing mix [0.5 µM of each hybridization probe (4 probes at a time), 6XSSPE, 0.01% Triton X-100] (all of the probes are listed in Table S2) was added over the gel and sealed with a frame seal chamber (HybriWell HBW2240, GRACE Bio-Labs). The slides were heated at 94°C for 6 min, followed by 58°C for 15 min. Free probes were removed by washing 2×4 min in wash buffer 1E. Following the first cycle, subsequent denaturation steps were performed at 65°C for sequential exon hybridization. Denaturation in the denaturing buffer will result in removal of the fluorophore-labeled exon probes and therefore is done before the annealing of the next set of exon probes.

All of the hybridization probes used for the polony assays are described in Table S2. The specificity of each probe was thoroughly tested by comparing its hybridization to control transcripts generated from control cDNA clones, e.g. with and without the exon in question. For example, the probe for exon 9B was hybridized against control RNAs containing or lacking the 9B exon. In addition, each was tested in combination with other probes with different fluorescent tags, to insure that no cross-hybridization or interference occurred. The hybridization and washing conditions were tested with all the probes to insure specificity, the polony slides were re-hybridized multiple times to insure reproducibility of the results and they were scanned after stripping off the probes to insure that no background fluorescence remained.

### Image acquisition and data analysis

All images of gels were acquired on a ScanArray 5000 instrument (Perkin Elmer) at 10 µm resolution using four lasers (635 nm, 532 nm, 488 nm, 594 nm). Gels were scanned after hybridization with labeled probes, as well as after probe stripping (to assess background signal). The myb-exon probed polony slide images were processed with Matlab to generate a composite. Co-localization of polonies allowed us to detect the exon context of the original molecule. A grid was overlaid on the images and a systematic random approach was performed to select fields for analysis. A total of 250–1500 polonies were counted per slide, and two slides were analyzed for each patient sample, for a total of 500–3000 polonies counted per patient sample, to provide estimates of c-*myb* splice variant expression. All counts were performed without knowledge of diagnosis or other clinical parameters.

### Sensitivity and linearity of the polony assay

PCR products containing unique alternative exons were cloned in an expression vector (pCDNA3; Invitrogen). To assess the alternative splice variant detection sensitivity of the polony assay, we performed the polony assay on different molar ratios of mixtures of wild-type c-*myb* transcript plasmid and the plasmid containing unique alternative exon 8A. To evaluate the quantitative ability of the polony assay, we serially diluted the wild-type plasmid from 5×10^−2^ ng/µL to 6×10^−4^ ng/µL, and used 1 µL from each dilution to perform the polony assay.

## Supporting Information

Figure S1 **Conservation of the c-myb 9S/10 splice variant.** (A) Comparison of the cryptic splice site in exon 9 from human, mouse and chicken and the predicted C-terminal amino acids in the corresponding c-Myb 9S/10 variant proteins. The 5′ splice site (gt) is shown underlined and in bold. All the predicted proteins terminate with two serine residues (in bold). (B) Expression of the c-myb 9S/10 variant in primary chicken and mouse bone marrow cells. RNA samples isolated from the chicken cell line HD3, primary chicken bone marrow or primary mouse bone marrow were subjected to RT-PCR using a forward primer spanning the unique 9S/10 splice junction and a reverse primer from exon 10. Nucleotide sequencing of the PCR products confirmed that they were the expected splice junction products from 9S/10 splice variants. The results suggest that the 9S/10 splice variant is conserved and expressed in human, mouse and chicken c-myb genes.
(TIF)

Table S1 Primers used for QRT-PCR using SYBR green detection.
(XLS)

Table S2 All primers used to perform polony amplification and exon-profiling.
(XLS)

Table S3 The expression levels of c-myb splice variants detected by polony assay in 13 pediatric pre-B ALL samples.
(XLS)
