# Supplementary figures and images for: Single Molecule Analysis of c-myb Alternative Splicing Reveals Novel Classifiers for Precursor B-ALL
Source: PLoS One. 2011 Aug 11;6(8):e22880. doi: 10.1371/journal.pone.0022880 (PMC3154906; doi:10.1371/journal.pone.0022880)

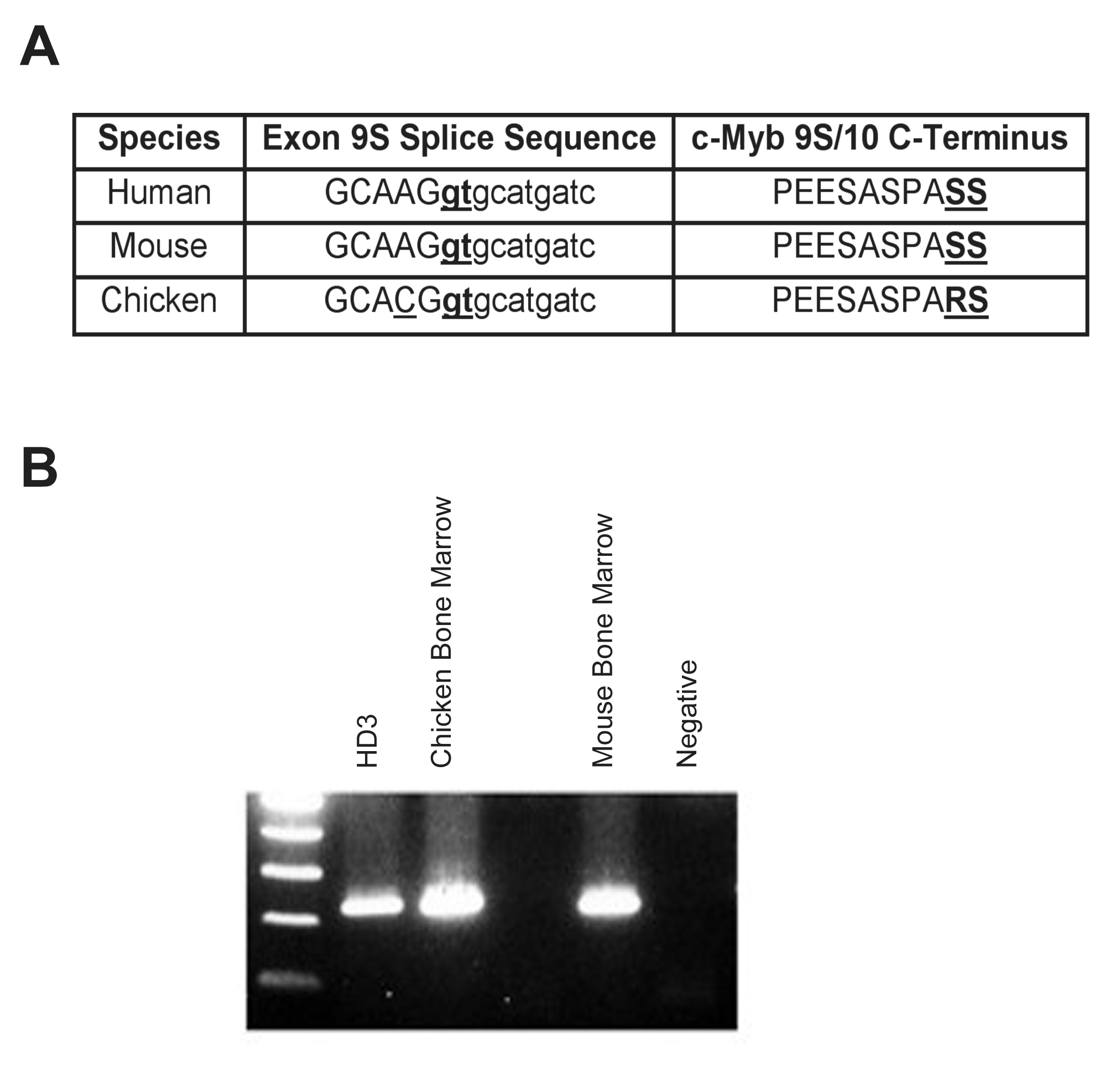

Supplement: Figure S1 — Conservation of the c-myb 9S/10 splice variant. (A) Comparison of the cryptic splice site in exon 9 from human, mouse and chicken and the predicted C-terminal amino acids in the corresponding c-Myb 9S/10 variant proteins. The 5′ splice site (gt) is shown underlined and in bold. All the predicted proteins terminate with two serine residues (in bold). (B) Expression of the c-myb 9S/10 variant in primary chicken and mouse bone marrow cells. RNA samples isolated from the chicken cell line HD3, primary chicken bone marrow or primary mouse bone marrow were subjected to RT-PCR using a forward primer spanning the unique 9S/10 splice junction and a reverse primer from exon 10. Nucleotide sequencing of the PCR products confirmed that they were the expected splice junction products from 9S/10 splice variants. The results suggest that the 9S/10 splice variant is conserved and expressed in human, mouse and chicken c-myb genes. (TIF) [file pone.0022880.s001.tif]
